# Patterns of Functional Gastrointestinal Disorders Among Children in Makkah City: A Single Institutional Experience

**DOI:** 10.7759/cureus.32224

**Published:** 2022-12-05

**Authors:** Hisham Alkhuzaei, Mohammed A Almatrafi, Wed Alqahtani, Rawan Alotaibi, Duaa Eid, Ebtehal Matar, Jawharah Tirkistani, Mohammad Nasim Khan, Khawlah Alharbi, Abdulwahab Telmesani

**Affiliations:** 1 Department of Pediatrics, Security Forces Hospital, Makkah, SAU; 2 Department of Pediatrics, Umm Al-Qura University, Makkah, SAU

**Keywords:** children, rome criteria, constipation, abdominal pain, functional gastrointestinal disorder

## Abstract

Background

Functional gastrointestinal disorders (FGIDs) are syndromes identified based on a group of symptoms defined according to the criteria of the Rome Foundation. The most commonly observed disorders among the pediatric population are functional abdominal pain disorders and functional constipation. This study aims to identify the patterns of FGIDs among children in Makkah, Saudi Arabia.

Methods

A retrospective cohort study was conducted at pediatric gastroenterology clinics from 2015 to 2019 in a tertiary centre in Makkah, Saudi Arabia. The FGID outcomes were compared with the patient's gender, age, and other characteristics using Statistical Package for the Social Sciences (SPSS, IBM Corp., Armonk, NY) software to analyze the data.

Results

One hundred and fifty-two participants met the inclusion criteria, with a mean age of 5.49 ± 3.27 and an average weight of 20.68 ± 12.15 kg. Male patients account for 59.2% of the total population. The prevalence of abdominal pain was 17.84%, while constipation was 50.93%. There was a statistically significant association between abdominal pain and independent variables such as family history (p=0.004) and age, particularly in older children (5-12 years; p=0.001). A statistically significant correlation was found between constipation with gender (p=0.032) and family history (p<0.001).

Conclusion

The prevalence of functional constipation and abdominal pain increased with age among children in Makkah City, with constipation being significantly more prevalent than functional abdominal pain. There is a significant relationship between age and family history with abdominal pain, whereas gender and family history are associated with a greater likelihood of constipation.

## Introduction

Functional gastrointestinal disorders (FGIDs) refer to a group of conditions characterized by persistent, recurrent gastrointestinal symptoms with no identifiable organic etiology [1]. The transition from Rome III to Rome IV criteria caused a substantial shift in the prevalence of FGID [2]. Based on Rome IV criteria, the prevalence of FGID in children with at least one FGID ranges from 22.2% in children aged 0-4 years to 21.8% in children aged 4-18 [3].

The pathophysiology of FGIDs is complex; nevertheless, several mechanisms are involved, including dysregulation of the brain-gut axis, dysbiosis, altered mucosal and immune functions, and visceral hypersensitivity [4,5]. Additionally, genetic predisposition and psychosocial factors may increase susceptibility to these disorders [4,5]. Recent research indicates that the biopsychosocial conceptual model best explains the pathophysiology of FGIDs [6]. This model defines FGIDs as the result of the interaction of genetic, environmental, and psychosocial factors that can alter gut physiology [6]. These interactions explain the variety of clinical presentations and severity among patients diagnosed with FGIDs [6].

The diagnosis of FGIDs is primarily clinical and based on the Rome IV criteria [4]. Rome IV classifies FGIDs into eight groups based on anatomical region and subclassifies each group according to symptoms [7,8]. The criteria identified 20 pediatric-related disorders, with a distinction made between younger (infant/toddler) and older (child/adolescent) children [7,8]. In the infant/toddler group, functional constipation and regurgitation were the most prevalent FGIDs [3]. In contrast, irritable bowel syndrome, functional constipation, and dyspepsia were most prevalent among children and adolescents [3].

In this study, we aim to evaluate the pattern, prevalence, and patient characteristics of functional constipation and abdominal pain among children attending the outpatient gastroenterology clinic at a tertiary hospital from 2015 to 2019 in Makkah, Saudi Arabia.

## Materials and methods

A retrospective observational cohort study was conducted from 2015 to 2019 at pediatric gastroenterology outpatient clinics in a single tertiary centre in Makkah, Saudi Arabia. This study's main objective is to evaluate the pattern, prevalence, and patient characteristics among children diagnosed with FGIDs. Pediatric patients up to the age of 12 years were assessed for eligibility to participate in this study. Children meeting the Rome IV case definition criteria for FGIDs were analyzed and included in the study. Exclusion criteria include patients over the age of 12, those who did not meet the case definition for FGIDs, and those with incomplete data in their medical records. The Institutional Review Board at the Security Forces Hospital approved this study (approval number: 0347-25022).

Study tool and data collection

Eligible patients' electronic medical records were reviewed and assessed for inclusion and exclusion criteria. Demographic data, clinical presentation, past family history of FGIDs and organic diseases, relevant laboratory tests, and imaging were collected and recorded in Excel-formatted data collection sheets. We compared the dependent variables of functional abdominal pain and constipation in children with concomitant characteristics, including gender, age, and family history.

Statistical analysis

After proper translation and coding, the data were entered into SPSS for analysis (IBM Corp., Armonk, NY). The frequency of cases identified for each category and sub-category were expressed in numbers (n) and percentages (%) of the total sample. Then χ2 (Fisher’s exact) test was used to measure any association between cases and different categorical variables (age group, gender, and family history). Binomial logistic regression was conducted to χ2 (Fisher’s exact) test significant variables to identify the risk associated. The odds ratio and 95% confidence interval were obtained from regression analysis. P-values <0.05 were considered significant.

## Results

The baseline characteristics of the patients are summarized in Table 1. A total of 152 participants were ultimately included in the present study. The mean age of the included children was 5.49 ± 3.27 years, and the mean weight was 20.68 ± 12.15 kg. Among the included patients, 90 (59.2%) were male children, while 62 (40.8%) were females. Moreover, most children were in the 5-12-year age group (48.7%), 1-3 years (30.3%), 3-5 years (17.1%), and 0-12 months (3.9%), respectively. Of the total patients, 11.8% had a family history of gastroenterology problems, 3.9% underwent endoscopy procedures, and 17.1% underwent ultrasound.

**Table 1:** Baseline characteristics of patients

	n (%)
Gender	
Female	62 (40.80%)
Male	90 (59.20%)
Age category	
0–12 months	6 (3.90%)
1–3 years	46 (30.30%)
3–5 years	26 (17.10%)
5–12 years	74 (48.70%)
Family history	
Yes	18 (11.80%)
No	134 (88.20%)
Ultrasound	26 (17.10%)
Endoscopy	6 (3.90%)

A total of 269 FGID patients visited the hospital during the 2015-2019 study period. Only patients who met ROME IV criteria were included in the study. Table 2 shows the prevalence of abdominal pain and constipation among our study population. Forty-eight (31.6%) patients were found to have recurrent abdominal pain, while 137 (90.1%) of the total patients had constipation.

**Table 2:** Functional gastrointestinal disease among children based on Rome IV guidelines

FGID	n (%)
Recurrent abdominal pain	48 (31.60%)
Constipation	137 (90.10%)

There was a statistically significant association between abdominal pain and individual independent variables, including age category and family history. Table 3 summarizes Fisher's exact/chi-squared p-values for each variable. There was no statistically significant association observed between abdominal pain and gender. It has been observed that the percentage of abdominal pain increases with age; 75% of the constipation patients were in the age group of 5-12 years old. In our study, abdominal pain was more likely to be seen in male patients than female patients. Of the total number of abdominal pain cases, 22.9% of patients had a family history.

**Table 3:** Association of abdominal pain and constipation with baseline characteristics

	Abdominal pain		p-value	Constipation		p-value
	Yes	No		Yes	No	
Age category						
0–12 months	0 (0%)	6 (5.8%)	<0.001	6 (4.4%)	0 (0%)	0.069
1–3 years	3 (6.3%)	43 (41.3%)		45 (32.8%)	1 (6.7%)	
3–5 years	9 (18.8%)	17 (16.3%)		24 (17.5%)	2 (13.3%)	
5–12 years	36 (75%)	38 (36.5%)		62 (45.3%)	12 (80%)	
Gender						
Female	22 (45.8%)	40 (38.5%)	0.39	52 (38%)	10 (66.7%)	0.032
Male	26 (54.2%)	64 (61.5%)		85 (62%)	5 (33.3%)	
Family history						
Yes	11 (22.9%)	7(6.7%)	0.004	11 (8%)	7 (46.7%)	<0.001
No	37 (77.1%)	97(93.3%)		126 (92%)	8 (53.3%)	

There was a statistically significant association between constipation and individual independent variables, including gender (p=0.032) and family history (p<0.001). Table 4 summarizes Fisher's exact/chi-squared p-values for each variable. There was no statistically significant association observed between constipation and age category. Constipation was found to be higher in 5-12 years of age. It has been found that male patients had a higher percentage (62%) of constipation than females (38%).

**Table 4:** Regression analysis of functional gastrointestinal disorder parameters with baseline characteristics of patients

Category	Abdominal pain		Category	Constipation	
	OR (95% CI)	p-value		OR (95% CI)	p-value
Age category			Gender		
1–3 years	Ref. category				
3–5 years	7.588 (1.830–31.462)	0.005	Female	Ref. category	
5–12 years	13.579 (3.867–47.682)	<0.001	Male	3.269 (1.059–10.096)	0.039
Family history			Family history		
Yes	4.12 (1.485–11.430)	0.007	Yes	0.1 (0.030–0.327)	<0.001
No	Ref. category		No	Ref. category	

Binomial regression analysis showed a statistical association between functional abdominal pain, age category, and the family history of patients. In addition to that, constipation was also found to be significantly associated with the gender and family history of the patients who participated in the study. Age group 5-12 years had a higher risk (OR = 13.579; CI = 3.867-47.682) of getting abdominal pain considering the 1-3 year age group as reference. Patients with an FGID family history had a 4.12-fold (OR = 4.12; CI = 1.485-11.430) increased risk of abdominal pain when compared to those without a family history. Compared to female patients, male patients had a higher chance of experiencing constipation (OR = 3.269; CI = 1.485-11.430). Patients who had a family history of FGID had a lower risk of getting constipation compared to others (OR = 0.100; CI = 0.030-0.327).

## Discussion

Functional disorders such as abdominal pain and constipation are common health problems in children; in some countries, FGID problems affect one-third of children [9]. Epidemiological studies assessing the prevalence of FGIDs in communities can aid in determining the depth of the problem and provide data to health planners in those communities [10]. The prevalence of FGIDs varies greatly among epidemiological studies, ranging from 1.6% to 41.2%. A meta-analysis published in 2015 estimated the prevalence of FGIDs to be around 13.5% [11]. This variation in prevalence may be attributable to methodological differences and diverse cultural, nutritional, genetic, and environmental factors [11]. Moreover, variations in the application of Rome criteria versions for the case definition of FGIDs may influence the prevalence; for instance, studies that used Rome III had a higher prevalence of FGID than Rome IV criteria [11,12].

The prevalence of abdominal pain increased linearly with age, increasing by approximately 6% per year [13]. Twenty-five percent of recurrent abdominal pain occurs between eight and twelve years of age [14]. This is in line with the findings of our study, which indicated that children aged 5-12 years are more likely to get abdominal pain.

One striking finding noted in our study was that male patients had a higher proportion of abdominal pain than female patients. This finding contradicts the results of a large meta-analysis that found a higher prevalence of functional abdominal pain disorders in female patients than in male patients (15.9% vs 11.5%) [11,15,16].

Our study found that functional constipation was the most common FGID among the study population. This finding is consistent with other studies published in numerous regions worldwide [10,17,18]. The prevalence of functional constipation in our study participants was 50.93%. This finding is higher than other studies in the scientific literature conducted in the United States and Greece [19]. The estimated prevalence of functional constipation in the literature ranges from 9.5% to 16% [17-19]. One possible explanation for this contradiction is the use of two distinct versions of the Rome criteria for patient allocation.

The prevalence of FGIDs was significantly higher among the parents of children with FGIDs (64% vs 30.7%) compared to the parents of children without FGIDs, highlighting that FGIDs are genetically determined and influenced by environmental factors [20]. In our study, family history was found to be a significant risk factor; our findings indicate that children with a family history of gastrointestinal disorders are more likely to develop functional constipation and functional abdominal pain. These results are consistent with those of previous studies [21,22]. According to a study published in 2012, those with a family history of constipation had considerably higher rates of functional constipation (49% vs 14.8%) [22].

Our present investigation had a few limitations. First, even though this study was conducted in a tertiary institution, a single institution may pose challenges regarding sample representation. Second, the retrospective design of this study makes it impossible to exclude recall bias. Third, some patients may have been missed because we are collecting data based on diagnosis codes.

## Conclusions

FGID is a common source of complaints among children and adolescents. Despite decades worth of clinical observation and research, it is still a challenging clinical entity with significant variations in its methodological approaches. According to the findings of this study, functional constipation was significantly more prevalent than functional abdominal pain among children in Makkah city. Furthermore, age and family history are significantly associated with functional abdominal pain, whereas gender and family history are significantly associated with functional constipation.
